# Toward an operative diagnosis in sepsis: a latent class approach

**DOI:** 10.1186/1471-2334-8-18

**Published:** 2008-02-19

**Authors:** Gisela D De La Rosa, Marta L Valencia, Clara M Arango, Carlos I Gomez, Alex Garcia, Sigifredo Ospina, Susana Osorno, Adriana Henao, Fabián A Jaimes

**Affiliations:** 1 Department of Internal Medicine, School of Medicine, Universidad de Antioquia, Medellín, Colombia; 2 Department of Internal Medicine, Section of infectious Diseases, Hospital Pablo Tobon Uribe, Medellín, Colombia; 3 Intensive Care Unit, Clínica Universitaria Bolivariana, Medellín, Colombia; 4 Epidemiology Department, Hospital Universitario San Vicente de Paul, Medellín, Colombia; 5 Department of Internal Medicine and Grupo Académico de Epidemiología Clínica, School of Medicine, Universidad de Antioquia, Medellín, Colombia

## Abstract

**Background:**

Recent data have suggested that 18 million of new sepsis cases occur each year worldwide, with a mortality rate of almost 30%. There is not consensus on the clinical definition of sepsis and, because of lack of training or simply unawareness, clinicians often miss or delay this diagnosis. This is especially worrying; since there is strong evidence supporting that early treatment is associated with greater clinical success. There are some difficulties for sepsis diagnosis such as the lack of an appropriate gold standard to identify this clinical condition. This situation has hampered the assessment of the accuracy of clinical signs and biomarkers to diagnose sepsis.

**Methods/design:**

Cross-sectional study to determine the operative characteristics of three biological markers of inflammation and coagulation (D-dimer, C-reactive protein and Procalcitonin) as diagnostic tests for sepsis, in patients admitted to hospital care with a presumptive infection as main diagnosis.

**Discussion:**

There are alternative techniques that have been used to assess the accuracy of tests without gold standards, and they have been widely used in clinical disciplines such as psychiatry, even though they have not been tested in sepsis diagnosis. Considering the main importance of diagnosis as early as possible, we propose a latent class analysis to evaluate the accuracy of three biomarkers to diagnose sepsis.

## Background

Sepsis is defined as host response to infection and it is an important cause of morbidity and mortality all around the world. Recent data have suggested that 18 million of new sepsis cases occur each year worldwide, with a mortality rate of almost 30% [1]. Incidence has risen due to aging of the population and the higher incidence of immunosuppressive conditions such as Acquired Immunodeficiency Syndrome (AIDS), chemotherapy for cancer, and use of invasive devices [2]. Sepsis costs on average US $ 22,000 per patient; in the United States US $16.7 billion are spent each year in sepsis care, which means a deep impact on financial resources [3].

The *surviving sepsis campaign*, a collaborative attempt from three major critical care organizations, issued a call for global action against sepsis. This Campaign pointed out sepsis diagnosis as a fundamental challenge [1]. There is no consensus on the clinical definition of sepsis and, because of lack of training or simply unawareness, clinicians often miss or delay this diagnosis. This is especially worrying; since there is strong evidence supporting that early treatment is associated with greater clinical success [4,5].

Ideally, "sepsis findings"-symptoms, signs, or biomarkers-should be compared with a "gold standard" test that is 100% sensitive and specific. This type of test rarely exists in clinical practice, but often there is a single test that is at least accurate enough to serve as a reference standard. Even this is not available for sepsis diagnosis, as microbiology is not enough sensitive and laboratory tests are not specific for using as reference standards. The lack of a reference test has hampered the assessment of the accuracy of clinical signs and biomarkers to diagnose sepsis. However, some clinical areas have dealt with this inconvenient. Psychiatrists make diagnoses without the benefit of laboratory, radiographies, or pathology reports. The lack of any reference standard in psychiatry has been overcome by using techniques that avoid the need for comparison with a single accurate test. These techniques can be broadly divided into latent class analysis (LCA) and Bayesian analysis [6]. LCA has been used widely in psychiatry as well as other disciplines [7-11] but, it has not been yet applied to the evaluation of the accuracy of clinical assessment in sepsis.

### The problem with the definitions

Over the last three decades, the syndrome now commonly referred to sepsis has alternately been called septicemia [12], sepsis syndrome [13] and, simply sepsis, the last definition described jointly with the closely related concept of systemic inflammatory response syndrome (SIRS) [14]. SIRS is considered to be present when patients present more than one of the following four clinical findings:

1) Body temperature > 38°C or < 36°C;

2) Heart rate > 90 beats min^-1^;

3) Hyperventilation evidenced by a respiratory rate > 20 breaths min^-1 ^or PaCo_2 _< 32 mm Hg;

4) White blood cell (WBC) count > 12,000 cells *μ*L^-1 ^or < 4,000 *μ*L^-1 ^or with > 10% immature forms.

A 1992 statement from the American College of Chest Physicians/Society of Critical Care Medicine (ACCP/SCCM) Consensus Conference, hypothesized that sepsis is a systemic response to infection; where infection is defined as an invasion process of a pathogenic or potentially pathogenic microorganisms to normally sterile tissue, fluids or body cavities [14]. According to this definition, a diagnosis of sepsis requires the presence of both infection and SIRS. Following the same model, sepsis with evidence of organic dysfunction would be characterized as severe sepsis; and sepsis with acute circulatory failure characterized by persistent hypotension unexplained by other causes, would be defined as septic shock.

However, as we showed in a recent work [15] and also as has been repeatedly pointed out by several experts [16,17], despite the fact that the SIRS definition considers that a systemic inflammatory response can be triggered by a variety of conditions (infectious and noninfectious), this particular combination of criteria are neither specific nor sensitive enough to be useful for making medical decision or to establish an accurate operative definition for the syndrome.

Nowadays, although no epidemiological evidence exists to support a change in the syndrome's definition (a systemic response to infection); it seems clear that the list of signs and symptoms of sepsis should be expanded to reflect clinical bedside experience. In this regard, the last *International Sepsis Definition Conference *[18] stated that a diagnosis of sepsis should be considered in the presence of a documented or suspected infection, concurrent with some markers of general illness, inflammation, hemodynamic disturbance, organ dysfunction or tissue perfusion abnormalities (Table 1.)

**Table 1:** Potential sepsis related markers (modified from reference 18)

*General Variables*
Temperature > 38.3°C or < 36°C
Heart rate > 90 beats min^-1^
Tachypnea (respiratory rate > 20 breaths min^-1 ^in adults)
Altered mental status

*Inflammatory variables*
WBC > 12,000 *μ*L^-1^, < 4,000 *μ*L^-1 ^or with > 10% immature forms
Plasma C-reactive protein > 2 SD above the normal value
Plasma procalcitonin > 2 SD above the normal value

*Hemodynamic variables*
Systolic blood pressure < 90 mm Hg or mean arterial blood pressure < 70 mm Hg
Mixed venous oxygen saturation > 70%
Cardiac index > 3.5 L*min^-1^*M^-23^

*Organ dysfunction variables*
PaO_2_/FIO_2 _< 300
Urine output < 0.5 mL*kg^-1^*hr^-1 ^or creatinine increase > 0.5 mg/dL
International normalized ratio (INR) > 1.5 or aPTT > 60 secs
Platelet count < 100,000 *μ*L^-1^
Plasma total bilirrubin > 4 mg/Dl

*Tissue perfusion variables*
Hyperlactatemia > 1 mmol/L
Decreased capillary refill or mottling

Notwithstanding the lack of conclusive criteria for sepsis, the definitions of severe sepsis (sepsis complicated by organ dysfunction) and septic shock (systolic blood pressure below 90 mm Hg or a reduction of > 40 mm Hg from baseline despite adequate volume resuscitation, in the absence of other causes for hypotension) remain undisputed. Unfortunately, this simple classification and range of definitions have strong limitations for precise characterization of sepsis, and mainly for the early staging of patients. Hence, the *International Sepsis Definitions Conference *[18], on the basis of ideas arisen from the *Fifth Toronto Sepsis Roundtable *[19], has proposed a classification scheme called -***PIRO***-. This staging system is aimed to be used for stratification of patients based on their ***P***redisposition, the type and extent of the ***I***nfection, the nature and magnitude of the host ***R***esponse, and the degree of associated ***O***rgan dysfunction.

The potential utility of this PIRO model lies in being able to discriminate morbidity arising from infection, which seems relatively mild, and morbidity arising from the host *response *to infection. Such a difference is critical since interventions that modulate the host response may impact adversely on the ability to contain an infection [19,20]. On the other hand, interventions that target exclusively the infection are unlikely to be beneficial if the morbidity is being driven by the host response. Besides, the same staging system may also provide a conceptual framework to improve the diagnosis process in sepsis. Specifically, appropriate characterization of *Infection *(injury) and *Response *(nature and magnitude of host responses) are *sine qua non *conditions to define, from a clinical point of view, the challenging spectrum of sepsis.

### Infection and host response in sepsis

There are several important issues in the pathogenesis of septic phenomenon. First, the host response, more than the nature or type of infection, appears as a critical determinant in patient prognosis. Second, monocytes and endothelial cells play a central role in initiating and perpetuating the host response. Third, sepsis is clearly associated with the simultaneous activation of the inflammatory and coagulation cascades, and most of their components are markers or mediators in the host response [19]. As corollary, the effort to defeat and eliminate pathogens may generate collateral damage on normal tissues, resulting in organ dysfunction and death [21].

The functional relationship between coagulation and inflammation within the pathogenic framework of sepsis has been recently dissected *in extensum *but, as yet, it is not completely understood [22-26]. In short, infection promotes coagulation via a large number of molecular and cellular mechanisms. The primary mechanism responsible for this pro-coagulant activity, however, may be the generation by monocytes and macrophages of pro-inflammatory cytokines, especially Interleukin 6 (IL-6), Interleukin 1*β*(IL-1*β*) and tumor necrosis factor (TNF). These cytokines, in turn, induce Tissue Factor (TF) expression in monocytes and endothelial cells, whereby extrinsic coagulation cascade is initiated [21,27]. At the same time, many clotting system components, such as thrombin, factor Xa and the TF-factor VIIa complex, working in conjunction with the inhibition of endogenous anticoagulants as antithrombin and activated protein C, act as boosters for the systemic inflammatory response in sepsis [28].

From this close interplay between inflammation and coagulation, which is a recognized way toward organ dysfunction and mortality, emerges the rationale to characterize the host response to infection. Indeed, the only effective complementary therapy for sepsis discovered on the last twenty years, Drotrecogin alfa (activated), a recombinant human activated protein C, is focused on modulation of the systemic inflammatory, pro-coagulant, and fibrinolytic responses [29]. Thus, although the clinical manifestations of inflammation and coagulation may be elusive, their biochemical features may be more consistent and constitute an attractive manner to characterize the syndrome. Three potential biomarkers have shown regular presence in systemic infections: C-reactive protein (CRP), procalcitonin (PCT), and D-dimer; the latter as an unspecific signal of coagulation activation [22,30,31]. So far, however, no large prospective studies support any of them as a single independent criterion for sepsis.

CRP is a typical acute-phase reactant and also is a largely studied marker of infection [30,32-34]. This protein binds to several polysaccharides present in bacteria, fungi and parasites, in the presence of calcium. These complexes activate the classical complement pathway, acting as opsonins and promoting phagocytosis. Recently, a calcium-dependent complex between CRP and very low density lipoprotein (VLDL) was also discovered in sera from critically ill patients, and it was associated with early changes in the activated partial thromboplastin time (aPTT) test, the development of disseminated intravascular coagulation (DIC), and the risks of mortality and sepsis [35,36]. CRP is predominantly synthesized by the liver and mainly in response to IL-6, with which exists a good correlation between serum levels. The secretion of CRP begins within 4–6 hours of the stimulus, doubling every 8 hours and peaking at 36–50 hours. After disappearance or removal of the stimulus, CRP falls rapidly, as it has a half-life of 19 hours. However, CRP can remain elevated, even for very long periods, if the underlying cause of the elevation persists. Only those interventions affecting the inflammatory process responsible for the acute phase reaction can change the CRP level. Changes may be very helpful in diagnosis as well as in monitoring response to therapy, as CRP levels are only determined by the rate of synthesis [30].

PCT is a 116-amino acid pro-hormone, which have been shown to be an extremely useful marker in sepsis, whether blood cultures are positive or negative; and in sepsis-like conditions such as severe burns, pancreatitis, inhalation injury, severe mechanical trauma after extensive surgery, and also in some infections of nonbacterial causation as severe malaria or systemic fungal infection [31,37-40]. Serum levels of PCT are frequently increased in sepsis patients, sometimes attain levels several thousand-fold normal, and these high levels often persist for long periods of time. Moreover, the levels often correlate positively with the severity of the condition and mortality [39,41]. In septic states PCT is produced throughout the body, and although experimentally is toxic to septic animals, it is not known how this polypeptide or its components might worsen the septic process. Furthermore, its normal physiologic actions, if any, are unknown [31].

As mentioned, activation of coagulation cascade is a common and early phenomenon in the development of sepsis, and this fact supports the use of anticoagulant treatments as potentially useful interventions. The final pathway in the coagulation system is characterized by the following reactions [42]: in the presence of thrombin, fibrinogen is cleaved to form fibrin monomers, which are subsequently stabilized by thrombin-activated factor XIII. Covalent cross-linkages in the D-domain region of fibrin produce an insoluble fibrin clot. The presence of the fibrin clot, in turn, triggers plasmin to lyse the clot as well as fibrinogen. Whereas fibrinogenolysis leads to fibrinogen-degradation products, lysis of the fibrin clot generates cross-linked fibrin-degradation products containing D-dimer. Generally, a finding of more than 500 ng of D-dimer per milliliter is considered abnormal, and such levels are present in virtually all of patients with sepsis [25,28]. In fact, the Drotrecogin trial showed that parallel with a decrease in serum IL-6, plasma D-dimer levels were significantly lower in patients in the intervention group than in patients in the placebo group on days 1 through 7 after the start of the infusion [29].

Diagnostic criteria in sepsis will be judged successful if clinicians regard them as an aid for decision-making at the bedside. Furthermore, any laboratory dependent criteria should use assays that are both, widely available and affordable everywhere. This diagnostic scheme requires sufficient sensitivity to identify most patients with the syndrome, while minimally sacrificing inevitable specificity [19,43]. According to conclusions addressed in the *International Sepsis Definition Conference *[18], "the operational definitions of sepsis may be refined and tested in the future as we increase our understanding of the immunologic and biochemical characteristics of these conditions." Therefore, a sound combination of inflammation and coagulation markers may provide the appropriate characterization to clinical suspicion in sepsis.

### Objectives

We aim to estimate the operative characteristics (sensitivity, specificity, predictive values and likelihood ratios) of three biological markers of inflammation and coagulation as diagnostic tests for sepsis, in patients admitted to hospital care with a presumptive infection as main diagnosis. Operative characteristics of individual markers will be estimated and three different combinations (CRP +DD, PCT+DD, CRP +PCT) will be evaluated as well.

## Methods/Design

### Study design

Cross-sectional study to determine the operative characteristics of a diagnostic test.

### Setting

Emergency Room (ER) service at the "Hospital Universitario San Vicente de Paúl" (Medellín, Colombia). This is a 550-bed, fourth level University Hospital, and is a reference institution for a region including approximately 3 million habitants. There are three intensive care unities (Surgical, Medical and Cardiovascular)

### Study population

#### Inclusion criteria

1. Patients hospitalized by the ER within 24 hours before admission to the study.

2. Aged 18 years or older

3. At least one of the following causes should be the main admission diagnosis to the hospital: a) any kind of infectious disease (confirmed or suspected), b) fever of unknown origin, c) delirium or any kind of encephalopathy of unknown origin, d) acute hypotension not explained by hemorrhage, myocardial infarction, stroke or heart failure, e) Adult Respiratory Distress Syndrome or f) multiple organ failure.

#### Exclusion criteria

1. Negative of the patients, their families or the physician to be part of the study.

2. Antimicrobial treatment before the beginning of the study received in other medical institution

3. Medical decision to treat the patient ambulatory or in other different institution within 24 hours of the study beginning.

The study population will be selected by an active search of possible candidates admitted to the ER. This search will be carried out permanently by research assistants with health care training. This study population widely defined but relevant for our research question, will allow us to study all the severity spectrum of this condition: patients with mild to severe infectious diseases, probably or confirmed cases by microbiology tests, and finally patients whom infectious causes will be discard as the responsible of hospital admission.

### Gold standard tests

Three gold standard tests will be considered in the analysis and their results will be determined without knowledge of the CRP, PCT and DD results. These gold standards will allow defining two study groups: patients with infection and/or sepsis (Positive by "Gold Standard") and patients without confirmed infection or sepsis (Negative by "Gold Standard"). The three gold standards are the following:

1. Presence of infection defined by microbiological confirmation

2. Clinical consensus defined by three experts with clinical practice in Internal medicine, Infectious diseases and Intensive care. This consensus will follow the criteria for nosocomial infection defined by the CDC; and the criteria proposed by the last International conference of sepsis definitions [18]. This criterion will be defined at the end of the first week of the patient admission to the study in order to have definitive results of microbiological tests. In this regard, this clinical "Gold Standard" let to define three groups: confirmed sepsis by microbiology, probably sepsis without microbiological evidence, and patients without sepsis.

3. Likelihood of sepsis diagnosis in the study population according to a latent class analysis (LCA).

### Procedures and data collection (Table 2)

**Table 2:** Schedule of evaluations and data collection

Evaluation	Screening	Admission to the study	Baseline evaluation	First Week	Daily during hospitalization and at 28-day
Clinical record review	**X**	**X**			
Informed consent		**X**			
APACHE II score			**X**		
Organ dysfunction evaluation			**X**		
Study tests			**X**		
Definition of infection (CDC)				**X**	
Gold Standards				**X**	
Microbiological tests			**X**		
Vital status					**X**

#### Definitions of the evaluations

##### Clinical record review

The main clinical diagnosis, the symptoms, the signs and the clinical data registered in the admission clinical record in the ER will be used in the screening process and the selection of the study population. The researchers will not have any participation in the decision about hospitalary or ambulatory treatment, as this responsibility is exclusively of the ER medical team.

##### Inform consent

Research assistants will describe the objective and the research procedures of the study to the potential participants. According to the local Institutional Review Board (Ethical Committee, Medical Research Center, Universidad de Antioquia), signed consent will be waived and oral approval will be obtained from all patients willing to participate.

##### APACHE II score

This is a recognized and validated indicator of severity and mortality risk in critical ill patients. This is an extremely useful tool to characterize the study population properly, and it will be determined by the research assistants in all patients admitted to the study within 24 hours after the hospital admission.

##### Organ dysfunction

The presence and severity of organ dysfunction is an important prognostic variable in critical ill patients. Also, the presence of organ dysfunction in a patient with suspected or confirmed sepsis is defined as severe sepsis. Hence, this variable is necessary to characterize the study population properly. The "*surviving sepsis campaign" *together with the "*Development of health care institute" *(Boston, MA, USA) have proposed a standard reference to some parameters that define this population. The presence of at least one of the following organ dysfunction variables constitutes a diagnosis of severe sepsis:

PaO_2_/FIO_2 _< 300

Urine output < 0.5 mL*kg^-1^*hr^-1 ^or creatinine increase > 0.5 mg/dL

Serum creatinin > 2 mg/dL

International normalized ratio (INR) > 1.5 or aPTT > 60 secs

Platelet count < 100,000 *μ*L^-1^

Plasma total bilirrubin > 4 mg/Dl

Tissue perfusion variables

Hyperlactatemia > 1 mmol/L

Metabolic acidosis (arterial pH<7.35 and PaCo_2 _normal o low values) non explained caused

Hypotension

Systolic blood pressure < 90 mm Hg or mean arterial blood pressure < 60 mm Hg. A 40 mm Hg descend respect previous values.

##### Study tests (CRP, PCT, DD)

CRP, PCT and DD will be carried out in all patients twice, at admission to the study and 24 hours after. These two measurements will allow to estimate a kinetic of the markers related to the evolution time of the disease, and to select the value that best reflects the chronology of the systemic response to the infection. CRP and DD are standardized and routine tests performed in the hospital's laboratory. The CRP is measured with a turbidimetry assay and its values are reported as milligrams per deciliter with a minimum value of 0.8 mg/dl. DD is measured with a modified ELISA test and its values are reported as nanograms per milliliter. Values lower than 500 ng/ml are considered normal with this technique.

The BRAHMS PCT LIA ^© ^is an immunoluminimetric assay used for determination of PCT in human serum and plasma. This consists of adding two specific monoclonal antibodies that link to the PCT in two different parts of the molecule-Calcitonin and catalacin parts. One of the antibodies is labeled by luminescence (trace), and the other one is fixed inside a little tube (Coated Tube System). During incubation both antibodies react to PCT forming a "sandwich complex", in this way the labeled antibody is linked with the tube surface. The signal magnitude of the luminescence is directly proportional to PCT concentration in the study sample. The analytic sensibility of the assays is about 0.1 ng/ml and the functional sensitivity -minimum measured value that diagnose with a maximum precision of 20% of variance interassay- is about 0.3 ng/ml. Lower values than 0.5 ng/ml are considered normal with this technique.

##### Infection definition

The presence of infection, defined according with clinical and microbiological criteria of the CDC definitions for nosocomial infections, will be part of one of the "Gold Standards" and will be determined by evaluation of three independent experts, who will be blinded of CRP, PCT, DD results.

##### Gold standards

Additionally to the microbiological confirmation and the expert consensus, a LCA will be used to estimate the likelihood of sepsis.

##### Blood cultures and other microbiological studies

Two or more blood samples in different sites will be obtained from all patients within 24 hours of admission and before the beginning of the antimicrobial treatment (In the case that the physician in charge decides to start empirical treatment). The samples will be processed by an automatized system for aerobic and anaerobic bacteria (Bactec 9240, Becton, Dickinson and Co, New Jersey) and the final report should include species and sensitivity to antimicrobials. Other biological samples as sputum, urine, pleural or peritoneal liquid or other kind of organic samples, will be required and processed according to clinical criteria for each patient and with the standard techniques of the institution.

##### Vital status

During all hospital treatment and at 28-day, the vital status will be observed in order to be able to estimate the global mortality and for subgroups in the study population. This determination will allow exploring the behavior of the diagnostic tests according with different severity levels, and also will allow evaluating their utility as prognostic markers.

## Discussion

### Measuring diagnostic accuracy where there is no "gold standard"

The performance of a diagnostic test is judged by how accurately the test result can identify a diseased or no diseased person. The true disease status is the "gold standard" against which a test should be compared. However, there are many conditions for which the definitive diagnosis is very difficult or expensive to establish. This is especially true for the diagnosis of a complex clinical condition as sepsis, in which even within the construct of "systemic response to infection" there is not a real "gold standard" against which the diagnostic criteria can be calibrated [18].

Psychological and social sciences have a long tradition in coping with primary study objects that are not directly observable. Constructs such as intelligence, fear or trust can only be measured indirectly. Inference proceeds by modeling the relationship between observable and latent variables in such a way that the parameters of interest are estimable from the implied relations between observable variables. When the unobservable variable is categorical, the term latent class analysis (LCA) applies [6]. In other words, LCA postulates the existence of an unobserved categorical variable that divides the population of interest into classes. Members of the population with a set of observed variables will respond differently depending on the latent class to which they belong. This technique can be applied to the problems related to diagnostic testing, with the unobserved categorical variable being "disease present" or "disease absent" [44]. The observed variables might typically be the results of three or more diagnostic tests, none of them being a gold standard. LCA can then be applied to estimate the proportions of patients in each latent class (that is, estimated to be diseased or free of disease), and the sensitivity and specificity of each diagnostic test. In summary, the goal of latent class analysis is to use the observed probabilities to estimate the unobserved ones.

The methodology for latent class analysis is one of the most active areas of biostatistics research and development in the last years [44-50], and specialized software is available for estimation procedures [10,11]. Considering the importance to have a diagnostic test that got a minimal proportion of false negative results, we have considered the sensitivity estimation to calculate the sample size. An adequate precision of the sensibility value gives enough power to detect significant values in the other operative characteristics. The number of the patients with the disease (NP) that is needed to give a sensibility estimation of 95%, with a 95% confidence interval +/- 3% is calculated with the following formula [51]:

NP=Z2α2×sensibility×(1−sensibility)(0,03×2)2NP=1,962×0,95×(0,05)(0,06)2=203

The NP (true positives and false negatives) is also determined by the prevalence (P) of the disease in the study population. Hence, the total of patients (TP) required for this research is a function of these two amounts:

$$TP=\frac{NE}{P}$$

According with the inclusion and exclusion criteria defined for our study population, the expected prevalence of microbiological confirmed sepsis is about 30% [3,15]. Therefore, the total participants we should recruited for an adequate sample size is 677 patients. Considering the necessity to carry out a pilot test to standardize PCT measurement, the total of patients recruited will be 700.

The cut points for the study tests (CRP, PCT, and DD) will be explored using Receptor Operative Characteristics (ROC) curves [52], using as classification criteria the gold standard clinical tests (presence of infection defined according with CDC modified criteria, and sepsis diagnosis defined as clinical consensus), but also using the presence of severe sepsis (organ dysfunction) and mortality. This will allow defining the cut points with the best sensibility without compromising the specificity significantly, but also to define useful values in clinical decisions.

A conventional method based in Bayes Theorem will be used to determine the operative characteristics of the tests and their different combinations against the referred gold standards [53]. 95% confidence intervals will be estimated for values of sensitivity, specificity, predictive values and likelihood ratios. STATA software (Stata Co, release 8.2, College Station, TX, USA, 2004) will be used for all analysis.

LCA is a statistical method developed to find subtypes of related cases (latent classes) that are inherent and implicit within multivariable categorical data [6]. A particularly application of the LCA is the evaluation of the accuracy of diagnostic tests when there is a lack of a "gold standard". In presence of at least three tests -CRP, PCT, DD- that can detect presence or absence of an illness, but without any of them that can determine certainly the condition; the LCA could be used to estimate the diagnostic accuracy of these tests. The traditional LCA assumes that results from the three tests in the same subject are independent within the real condition of illness [44]. In other words, the conditional or local independence assumption affirms that inside each latent class (sepsis or no sepsis); each result of a test is statistically independent of the result of the other one. If the effect to belong to a latent condition of sepsis would be removed, the effects to the CRP, PCT and DD would have a completely random distribution in the study population. However, in many clinical situations this independence assumption is less likelihood and in some cases is extremely difficult to verify. In our study it is probably that PCT and CRP values are related directly each other within an inflammation process.

This local independence assumption can be relaxed or controlled introducing a random effect through a continuous latent variable [47]. In the LCA with random effects, it is assumed that the result of diagnostic tests is controlled by two mechanisms or factors. The first one is the real condition of the illness (*δ*) and the second one is the biological individual process in the patient or the technical characteristics of the test. In this regard, the model introduces another latent variable (t) that summarizes or represents the subject or the diagnostic test attributes that are not explained by the real condition of the illness. Hence, it is assumed that the results of the different diagnostic tests are independent, conditionals in *δ *and t. Similarly, it is assumed that *t *is distributed according to a standard normal distribution and that the probability to have a positive result from the test (Pr Y = 1), given *δ*, is a monotonic function of *t*, this is represented by the following equation:

Pr(*Y*_*i *_= 1|*δ*, *t*) = Φ(*a*_*iδ *_+ *b*_*δ*_*t*)

In this equation, "Φ", represents the function of the accumulated density of the normal distribution, and the *a *and *b *terms are the parameters. The positive rate of the test, conditionally in *δ*, is simply the mean value over *t*. The estimators of maximum verisimilitude for sensibility and specificity of each test could be obtained with an integral that uses an iteration algorithm such as the EM or the Newton-Rapson method [46,47]. All the previous analysis and convergence procedures to the latent class estimation will be carried out with the statistical software Latent GOLD 4.0 (Statistical Innovations, Belmont, MA, USA)

The existence of two clinical "gold standards", as previously described, will allow us to compare them against the LCA results. In this way it is possible to analyze the differences and similarities when sepsis diagnosis is defined by LCA comparing by clinical consensus or simple infection criteria. Thus, the strong biological assumption of a crosstalk between inflammation and coagulation in sepsis, and a sensible mathematical model of the latent diagnostic classification, provide a unique opportunity to understand a relevant clinical and public health problem.

## Competing interests

The author(s) declare that they have no competing interests.

## Authors' contributions

GD and FJ conceived the study, participated in its design and drafted the manuscript. MV, CA, CG, AG, SO, AH, and SO participated in the design. All authors read and approved the final manuscript.

## Pre-publication history

The pre-publication history for this paper can be accessed here:


